# Supplementary material for: Racial discrimination, self-efficacy, and oral health behaviours in adolescents
Source: PLoS One. 2023 Aug 15;18(8):e0289783. doi: 10.1371/journal.pone.0289783 (PMC10426965; doi:10.1371/journal.pone.0289783)
Supplement: S1 Checklist — (DOCX) [file pone.0289783.s001.docx]

STROBE Statement—checklist of items that should be included in reports of observational studies

|  | Item No. | Recommendation | Page  No. | Relevant text from manuscript |
| --- | --- | --- | --- | --- |
| **Title and abstract** | 1 | (*a*) Indicate the study’s design with a commonly used term in the title or the abstract | 2 | A cross sectional study of adolescents aged 12 to 18 years |
|  |  | (*b*) Provide in the abstract an informative and balanced summary of what was done and what was found | 2 | Of 252 participants, mean (SD) age was 14 (1.8) years old. 60% were female, 81% were born in Canada, 56% identified themselves as White, and 20% perceived discrimination. Mean score of all task-specific self-efficacies were significantly different within respective oral health behaviour categories (P-value <0.001). Of demographics, age and ethnicity (White) were significantly associated with discrimination (OR=1.25: 95% CI; 1.06 – 1.48 and OR=0.29: 95% CI; 0.15 -0.55, respectively). Perceived discrimination was positively associated with higher sugar consumption and mediate the association between diet self-efficacy and adolescent’s dietary behaviour |
| Introduction | | | |  |
| Background/rationale | 2 | Explain the scientific background and rationale for the investigation being reported | 4 | The experience of discrimination may moderate the association of self-efficacy and oral health behaviours. According to Hansen (2015), discrimination has been linked to various health impairment such as chronic muscle pain, diabetes, and metabolic syndrome. The relationship with mediation effect of discrimination has not yet been fully investigated in oral health. Therefore, the aims of this study were to examine the mediation effect of discrimination on the association of self-efficacy and oral health behaviours among adolescents. |
| Objectives | 3 | State specific objectives, including any prespecified hypotheses | 4 | the aims of this study were to examine the mediation effect of discrimination on the association of self-efficacy and oral health behaviours among adolescents. |
| Methods | | | |  |
| Study design | 4 | Present key elements of study design early in the paper | 2 | A cross sectional study of adolescents aged 12 to 18 years |
| Setting | 5 | Describe the setting, locations, and relevant dates, including periods of recruitment, exposure, follow-up, and data collection | 4, 5 | University of Alberta dental clinic in 2021.  A questionnaire consisting of three sections were completed by adolescents. |
| Participants | 6 | (*a*) *Cohort study*—Give the eligibility criteria, and the sources and methods of selection of participants. Describe methods of follow-up  *Case-control study*—Give the eligibility criteria, and the sources and methods of case ascertainment and control selection. Give the rationale for the choice of cases and controls  *Cross-sectional study*—Give the eligibility criteria, and the sources and methods of selection of participants | 4 | Aged 12-18-year-old who could understand English and had no significant physical or mental disabilities were included in the study. Affordable costs and high volumes of visits were the reasons to choose the University dental clinic to recruit participants |
|  |  | (*b*) *Cohort study*—For matched studies, give matching criteria and number of exposed and unexposed  *Case-control study*—For matched studies, give matching criteria and the number of controls per case |  |  |
| Variables | 7 | Clearly define all outcomes, exposures, predictors, potential confounders, and effect modifiers. Give diagnostic criteria, if applicable | 6 | Outcome variables included oral health’s behaviour. In this study, discrimination and self-efficacy score were predictors of the model and the confounding factors included age, gender, location of birth, ethnicity, and income. |
| Data sources/ measurement | 8* | For each variable of interest, give sources of data and details of methods of assessment (measurement). Describe comparability of assessment methods if there is more than one group |  |  |
| Bias | 9 | Describe any efforts to address potential sources of bias. | 4 | Affordable costs and high volumes of visits were the reasons to choose the University dental clinic to recruit participants |
| Study size | 10 | Explain how the study size was arrived at | 5 | The power of study based on type 1 error was reasonable based on sample size. |

Continued on next page

| Quantitative variables | 11 | Explain how quantitative variables were handled in the analyses. If applicable, describe which groupings were chosen and why | 6 | Percentages represented categorial variables, and continuous variables were identified by means, standard deviations, and ranges. Univariate analyses were performed to examine the impact of any demographic factors on the outcomes of interest. |
| --- | --- | --- | --- | --- |
| Statistical methods | 12 | (*a*) Describe all statistical methods, including those used to control for confounding | 6 | Mediation and hierarchal logistic regression analyses were conducted to find if discrimination had any mediation effect on associations between task-specific self-efficacy and general self-efficacy and the respective oral health behaviour. |
|  |  | (*b*) Describe any methods used to examine subgroups and interactions |  |  |
|  |  | (*c*) Explain how missing data were addressed | 5 | Those variables with more than 10% missing were not included in multivariant analysis |
|  |  | (*d*) *Cohort study*—If applicable, explain how loss to follow-up was addressed  *Case-control study*—If applicable, explain how matching of cases and controls was addressed  *Cross-sectional study*—If applicable, describe analytical methods taking account of sampling strategy |  |  |
|  |  | (*e*) Describe any sensitivity analyses |  |  |
| Results | | | | |
| Participants | 13* | (a) Report numbers of individuals at each stage of study—eg numbers potentially eligible, examined for eligibility, confirmed eligible, included in the study, completing follow-up, and analysed |  |  |
|  |  | (b) Give reasons for non-participation at each stage |  |  |
|  |  | (c) Consider use of a flow diagram |  |  |
| Descriptive data | 14* | (a) Give characteristics of study participants (eg demographic, clinical, social) and information on exposures and potential confounders | 6 | A total of 252 participants were included in the analyses with mean (SD) age of 14 (1.8) years old. Sixty percent of participants were girl and age were not statistically different between males and females (p-value > 0.05). Eighty-one percent of participants were born in Canada and 56% were self-identified as White (Caucasian). Of those participants who responded, 65% of mothers had college or university education and 61% had dental insurance (insurance variable has 55% missing data). Participants’ demographics are presented in **Table 1**. About 30% of participants reported their oral health as not good and about 20% reported some experience of discrimination. |
|  |  | (b) Indicate number of participants with missing data for each variable of interest |  |  |
|  |  | (c) *Cohort study*—Summarise follow-up time (eg, average and total amount) |  |  |
| Outcome data | 15* | *Cohort study*—Report numbers of outcome events or summary measures over time |  |  |
|  |  | *Case-control study—*Report numbers in each exposure category, or summary measures of exposure |  |  |
|  |  | *Cross-sectional study—*Report numbers of outcome events or summary measures |  |  |
| Main results | 16 | (*a*) Give unadjusted estimates and, if applicable, confounder-adjusted estimates and their precision (eg, 95% confidence interval). Make clear which confounders were adjusted for and why they were included | 6, 8 | Significant correlations were found among general and all task-specific self-efficacies. (**Table 2**). In the adjusted analysis for the participants’ demographics, odds ratio (OR) of brushing more than twice per day increased by any unit increase in toothbrushing self-efficacy (OR=1.30; 95% CI: 1.13- 1.51). For the diet, with any unit increase in diet self-efficacy, the risk of consuming sugar decreased 9% (OR=0.89; 95% CI: 0.83 -0.95). In addition, participants with higher dental visit self-efficacy had 11% higher chance of a dental checkup (OR=1.11; 95% CI; 1.03 – 1.20). When examining general self-efficacy with oral health behaviour, only toothbrushing frequency was significantly associated with GSE (OR=1.05: 95%CI; 1.01 – 1.09). |
|  |  | (*b*) Report category boundaries when continuous variables were categorized |  |  |
|  |  | (*c*) If relevant, consider translating estimates of relative risk into absolute risk for a meaningful time period |  |  |

Continued on next page

| Other analyses | 17 | Report other analyses done—eg analyses of subgroups and interactions, and sensitivity analyses | 163-172 | Significant mediation effect of perceived discrimination on the relationship of diet self-efficacy and respective oral health behaviour was observed (P-value=0.001). While the beta coefficient of direct association of diet self-efficacy and respective oral health behaviour was -0.16, total beta coefficient (direct and in-direct) was - 0.19. Indirect effect was statistically significant (**Figure 1**). In the univariate analyses, of all participants’ demographic characteristics, age and ethnicity had significant associations with discrimination (OR=1.25: 95% CI; 1.06 – 1.48 and OR=0.29: 95% CI; 0.15 -0.55, respectively) (**Table 4**). Findings illustrated that in those participants experiencing discrimination, risk of sugar consumption was 2.7 times higher (OR=2.67; 95% CI; 1.40 – 5.08) than in those who did not report racial discrimination. |
| --- | --- | --- | --- | --- |
| Discussion | | | | |
| Key results | 18 | Summarise key results with reference to study objectives | 272-279 | General and oral health-related self-efficacy were associated with oral health-related behaviours such as toothbrushing and sugar-intake among adolescents. Perceived discrimination could significantly medicate association of diet subclass of self-efficacy with its respective behaviour. Thus, assessment of self-efficacy is beneficial and useful in dental practice to identify those with low self-efficacy. Behavioural interventions to increase patient’s self-efficacy may buffer the negative impact of perceived discrimination leading to better adherence of adolescents to healthy behaviours and, consequently, better oral health outcomes. |
| Limitations | 19 | Discuss limitations of the study, taking into account sources of potential bias or imprecision. Discuss both direction and magnitude of any potential bias. | 256-261 | First, there is some selection bias with the participants in the study. All participants were recruited from the University clinic that reduces the ability to extrapolate our findings to the general population. Second, participants seeking dental care have higher motivation or better access to oral health resources that may distort true associations. Moreover, assessments of oral health behaviour were self-reported, which may have resulted in overestimation or underestimation |
| Interpretation | 20 | Give a cautious overall interpretation of results considering objectives, limitations, multiplicity of analyses, results from similar studies, and other relevant evidence | 272-279 | General and oral health-related self-efficacy were associated with oral health-related behaviours such as toothbrushing and sugar-intake among adolescents. Perceived discrimination could significantly medicate association of diet subclass of self-efficacy with its respective behaviour. Thus, assessment of self-efficacy is beneficial and useful in dental practice to identify those with low self-efficacy. Behavioural interventions to increase patient’s self-efficacy may buffer the negative impact of perceived discrimination leading to better adherence of adolescents to healthy behaviours and, consequently, better oral health outcomes. |
| Generalisability | 21 | Discuss the generalisability (external validity) of the study results 267-270 |  | The results of this study are not generalizable to the whole adolescent population because we recruited the participants from dental clinics, therefore, could have a more favourable attitude towards oral health and dental visits and less barrier to access dental care. |
| Other information | |  | | |
| Funding | 22 | Give the source of funding and the role of the funders for the present study and, if applicable, for the original study on which the present article is based | 282-283 | The authors received no financial support. |

*Give information separately for cases and controls in case-control studies and, if applicable, for exposed and unexposed groups in cohort and cross-sectional studies.

**Note:** An Explanation and Elaboration article discusses each checklist item and gives methodological background and published examples of transparent reporting. The STROBE checklist is best used in conjunction with this article (freely available on the Web sites of PLoS Medicine at http://www.plosmedicine.org/, Annals of Internal Medicine at http://www.annals.org/, and Epidemiology at http://www.epidem.com/). Information on the STROBE Initiative is available at www.strobe-statement.org.
