# Supplementary material for: Racial discrimination, self-efficacy, and oral health behaviours in adolescents
Source: PLoS One. 2023 Aug 15;18(8):e0289783. doi: 10.1371/journal.pone.0289783 (PMC10426965; doi:10.1371/journal.pone.0289783)
Supplement: S1 Table — (DOCX) [file pone.0289783.s002.docx]

**Part 1: Demographic Data (Adolescents)**

1. Date of birth: ____/____/_______ (mm/dd/yyyy)
2. Which grade are you in? Grade ______
3. Sex Assigned at birth: □ Male □ Female □ intersex □ prefer not to disclose
4. Were you born in Canada? □ Yes □ No
5. When did your family arrive in Canada? _____________ (YEAR) □ I don’t know
6. What is your family race/ethnicity background? _______________________
7. Have you ever been treated unfairly or discriminated against because of your race?

□ Never □ little bit □ fair bit □ lots of times

1. Are you living with? □ Both parents □ Single parents □ Other, specify ___________
2. What is the size of your family? Number of children _____ Number of adults _____
3. What is your mother’s education level?

□ Less than high school □ High school □ College/University □ I don’t know

1. What is your father’s education level?

□ Less than high school □ High school □ College/University □ I don’t know

1. Do you have a dental coverage? □ Yes □ No □ I don’t know

**Part 2: Oral Health Behavior**

1. How do you think your oral health is?

□ Very good □ Good □ Fair □ Not good □ Poor

1. Do you know if you have any cavities or gum disease?

□ Yes □ No □ I don’t know

1. When was the last time you visited a dentist or dental hygienist?

□ Within the last 12 months □ Over one year □ Never had one

1. If you had a dental visit, what was (were) the reason(s)? (Check all that apply)

□ Regular check-up □ Non-urgent dental problems □ Urgent dental problems

□ Others (please specify)______________

1. How many times a day you brush your teeth?

□ Less than once a day □ Once □ Twice □ More than twice

1. How often do you consume foods or drinks (for example juice, pop, candies, cookies, etc.) high in sugar?

□ Never □ Less often than everyday □ Once a day □ Twice day

□ Three times a day or more often

**Part 3: Self-Efficacy Scale for Oral Health (SEOH) and GSE**

| **Questions** | **Not confident** | **Less confident** | **Slightly confident** | **Moderately confident** | **Completely**  **confident** |
| --- | --- | --- | --- | --- | --- |
| **Self-efficacy for tooth brushing** | | | | | |
| 1. I brush my teeth as instructed. | 1 | 2 | 3 | 4 | 5 |
| 1. I brush my teeth carefully and thoroughly. | 1 | 2 | 3 | 4 | 5 |
| 1. I brush the border between the teeth and gum. | 1 | 2 | 3 | 4 | 5 |
| 1. I move the tooth brush with a short quick motion. | 1 | 2 | 3 | 4 | 5 |
| 1. I take time to brush my teeth carefully. | 1 | 2 | 3 | 4 | 5 |
| **Self-efficacy for dietary habits** | | | | | |
| 1. I try not to spend too much time eating. | 1 | 2 | 3 | 4 | 5 |
| 1. I eat my meals at fixed times during the day. | 1 | 2 | 3 | 4 | 5 |
| 1. I try to eat a well-balanced diet. | 1 | 2 | 3 | 4 | 5 |
| 1. I try not to eat or drink right before bed. | 1 | 2 | 3 | 4 | 5 |
| 1. I try not to eat too much sweets. | 1 | 2 | 3 | 4 | 5 |
| **Self-efficacy for dental visits** | | | | | |
| 1. I go to dentist for treatment of tooth decay of gum disease. | 1 | 2 | 3 | 4 | 5 |
| 1. I cooperate with my dentist during the treatment. | 1 | 2 | 3 | 4 | 5 |
| 1. I visit my dentist regularly even after treatment is completed. | 1 | 2 | 3 | 4 | 5 |
| 1. I go for regular check-ups even if I am busy. | 1 | 2 | 3 | 4 | 5 |
| 1. I go for regular check-ups even if my mind is relaxed. | 1 | 2 | 3 | 4 | 5 |
| **General Self-efficacy** | | | | | |
| 1. I can always manage to solve difficult problems if I try hard. | 1 | 2 | 3 | 4 | 5 |
| 1. If someone opposes me, I can find a way to get what I want. | 1 | 2 | 3 | 4 | 5 |
| 1. It is easy for me to stick my aims and accomplish my goals. | 1 | 2 | 3 | 3 | 5 |
| 1. I am confident that I can deal efficiently with unexpected events. | 1 | 2 | 3 | 4 | 5 |
| 1. Thanks to my resourcefulness, I know how to handle unforeseen situations. | 1 | 2 | 3 | 4 | 5 |
| 1. I can solve most problems if invest the necessary efforts. | 1 | 2 | 3 | 4 | 5 |
| 1. I can remain calm when facing difficulties because I can rely on my coping abilities. | 1 | 2 | 3 | 4 | 5 |
| 1. When I am confronted with a problem, I can usually find several solutions. | 1 | 2 | 3 | 4 | 5 |
| 1. If I am in trouble, I can usually think of a solution. | 1 | 2 | 3 | 4 | 5 |
| 1. I can usually handle whatever comes my way. | 1 | 2 | 3 | 4 | 5 |
