# Supplementary material for: Racial discrimination, self-efficacy, and oral health behaviours in adolescents
Source: PLoS One. 2023 Aug 15;18(8):e0289783. doi: 10.1371/journal.pone.0289783 (PMC10426965; doi:10.1371/journal.pone.0289783)
Supplement: Attachment — Submitted filename: Rebuttual letter-PLOS-One Journal.docx [file pone.0289783.s003.docx]

RE: “Discrimination, self-efficacy and oral health behaviours in adolescents”

Dear. Dr. Chenette,

I would like to thank you and your reviewers for taking the time to consider our manuscript for publication in PLoS One Journal. The authors would like to thank the reviewers for providing their constructive feedback so that we may strengthen our manuscript even further.

Below, you will find our responses for each comment made by the reviewers followed by the revised text if applicable. We also revised manuscript accordingly. Please let us know if you need any other changes or clarification.

Sincerely,

Maryam Amin, DMD, MSc, PhD

University of Alberta, School of Dentistry

5-513 Edmonton Clinic Health Academy

11405 - 87 Ave NW

Edmonton, AB, T6G 1C9

Email Address: [maryam.amin@ualberta.ca](mailto:maryam.amin@ualberta.ca)

1. Please ensure that your manuscript meets PLOS ONE's style requirements, including those for file naming. The PLOS ONE style templates can be found at 
<https://journals.plos.org/plosone/s/file?id=wjVg/PLOSOne_formatting_sample_main_body.pdf> and 
<https://journals.plos.org/plosone/s/file?id=ba62/PLOSOne_formatting_sample_title_authors_affiliations.pdf>

**Response:** The manuscript was reviewed to ensure the requirements are met.

2. You indicated that you had ethical approval for your study. In your Methods section, please ensure you have also stated whether you obtained consent from parents or guardians of the minors included in the study or whether the research ethics committee or IRB specifically waived the need for their consent.

**Response:** Thank you for the comment. We have added signed consent statement in the manuscript.

**Revised text:** Signed consent and assent forms were obtained from the guardians and adolescents in advance to collecting data from participants.

3. In your Data Availability statement, you have not specified where the minimal data set underlying the results described in your manuscript can be found. PLOS defines a study's minimal data set as the underlying data used to reach the conclusions drawn in the manuscript and any additional data required to replicate the reported study findings in their entirety. All PLOS journals require that the minimal data set be made fully available. For more information about our data policy, please see <http://journals.plos.org/plosone/s/data-availability>.

**Response**: Data is available upon request.

Review 1

General comments:

The authors demonstrated that perceived racial discrimination is related to the deterioration of diet self-efficacy and an increase in the intake of sugar-containing food. Potentially, the topic of the manuscript is interesting; however, there are some issues that should be addressed in the manuscript.

Abstract:

Please describe the study setting.

- **Response**: Thank you for your comment. We have added “Outpatient dental clinics” to the abstract.
- **Revised text**: **Methods**: A cross sectional study of adolescents aged 12 to 18 years who were recruited from outpatient dental clinics were asked to complete a questionnaire consisting of: demographics (12 items), oral health behaviours (7 items), general self-efficacy (10 items) and self-efficacy for self-care (SESS, 15 items).

Please explain regarding path analysis.

- **Response**: Thank you for your comment. Using pathway analyses, the relationship between oral health behaviours, self-efficacy and discrimination was explored.
- **Revised text:** Perceived discrimination was assessed if the adolescent had ever been treated unfairly based on their race. Using pathway analyses, the relationship between oral health behaviours, self-efficacy, and discrimination was explored. Mediation and hierarchal logistic regression analyses were conducted.

Introduction:

P10L87-88: Authors argued that the relationship with mediation effect of discrimination has not yet been fully investigated in oral health. I agree your argument. However, many studies on the relationship between oral health and discrimination were reported, for instance, Racial Inequalities in Oral Health. (J Dent Res. 2018 Jul;97(8):878-886), Intersectionality, racial discrimination and oral health in Australia. (Community Dent Oral Epidemiol. 2021 Feb;49(1):87-94), Perceived racial discrimination partially mediates racial-ethnic disparities in dental utilization and oral health. (J Public Health Dent. 2022 Mar;82 Suppl 1(Suppl 1):63-72, Racial and oral health equity in dental school curricula. (J Public Health Dent. 2022 Mar;82 Suppl 1(Suppl 1):114-122).
Authors should cite the previous reports regarding the relationship, and the author should clearly explain in their research why they conducted the study by stating what has already been discovered and what remains unknown based on previous research.

- **Response:** Thank you for your comment. The following literatures were added to manuscript and cited.
- **Revised text:**
- Previous research has also determined the role of discrimination as a psychosocial stressor that contributes to racial inequalities in oral health outcomes. Furthermore, it highlights the importance to go beyond the individual-level factors like socioeconomic status and genetic makeup and to consider wider sociohistorical processes such as systematic racism, economic exploitation, social stigmatization, and political marginalization in racial health outcomes [21]. Similarly, the study conducted by Schuch et al. with Australian adults demonstrated that discrimination negatively affected oral health outcomes; however, the impacts were more pronounced in socially marginalized groups [22]. Furthermore, after controlling for other risk factors such as income, education, age, sex, Marital status, employment status, and type of social insurance, Hispanic individuals with perceived discrimination were less likely to visit a dentist and more likely to have tooth loss compared to non-Hispanic white individuals [23]. Another study conducted by Christinia et al. also indicated the importance of addressing racism and its physiological impacts on oral health in dental education in order to promote oral health equity, regardless of ethnic backgrounds [24]. Yet, the mediation effect of discrimination has not been fully investigated in oral health.

Methods:

Explain using path analysis.

- **Response:** Thank you for your comment. Further explanation of path analysis has been provided.
- **Revised text:** Using AMOS, a visual program for path analysis, the relationships between self-efficacy, discrimination, and oral health behaviors were investigated to find whether discrimination had any mediation effect on the association between self-efficacy and oral health behaviors or self-efficacy mediated the other two components association.

Results:

Please describe the participant’s rate.

- **Response:** We used a convenience sampling approach and almost all adolescents approached completed the survey. Therefore, the participants rate was about 90%.
- **Revised text:**  A total of 252 participants were included in the analyses with mean (SD) age of 14 (1.8) years old. We used a convenience sampling approach and almost all adolescents approached completed the survey. Therefore, the participants rate was about 90%. Sixty percent of participants were girl and age were not statistically different between males and females (p-value > 0.05).

Please provide the Cronbach’s alpha for each scale.

- **Response:** Thank you for the comment. The Cronbach’s alpha for each scale was added.
- **Revised text:** Internal consistency coefficient of self-efficacy items was 0.90. Cronbach's alpha was also greater than 0.80 suggesting that the scales were homogenous.

Table 1 should describe the characteristics of each group of participants divided based on whether they have experienced discrimination or not, with statics analysis.

**Response**: In this study, we presented participants’ characteristics and survey results for the whole cohort and considered perceived discrimination as one variable of interest. Mediation effect of the perceived discrimination was then incorporated in two stage models (with and without discrimination variable). In other words, we did not intend to compare the two cohorts with or without perceived discrimination. More explanation was added to the text to present the most important characteristic of participants reported discrimination.

**Revised text:** Of all participants, 52 individuals (61% female) with mean (SD) age of 15 (2) years reported discrimination. This group was on average one year older than those who did not report discrimination (P-value <0.05). No statistically significant difference was found for sex and insurance coverage between the two groups (P-value>0.05).

Table 3 Please explain mean of the symbol in footnote.

- **Response:** Mean of the symbol was explained in the footnote
- **Revised text**:

| Yes | 52 (20) | 18.4* | 16.3* | 18.5 | * sig | 36.7 | > 0.05 |
| --- | --- | --- | --- | --- | --- | --- | --- |
| No | 200 (80) | 20 | 14.5 | 19.5 |  | 38.2 |  |
| *Statistically significant p-value <0.05, NA: Not applicable | | | | | | | |

Figure 1. Did diet self-efficacy have negative influence on discrimination? Please recheck the model for path analysis, and perform goodness-of-fit test for the model.

**Response:** Thank you for the comment. We rechecked the model and provided the details in the manuscript.

**Revised text**: Mediation between perceived discrimination, general and task-specific self-efficacy, and oral health behaviours were tested using IBM® SPSS® Amos. CMIN/DF for the default model was 7.2, providing close to reasonable goodness of fit (CMIN = Chi-square value) (DF = Degree of Freedom).

Table 5. Please provide data on the goodness of fit of each model in regression analysis.

- **Response:** Thank you for your response. Data on the goodness of fit of each model in the regression analysis was included.
- **Revised text:** In adjusted hierarchal logistic regression analyses, chi-square for Hosmer and Lemeshow test for the final model was 3.58 and statistically non-significant indicating that model fitted the data reasonably well.

Discussion:

L253-257. I agree with the discussion. However, the authors should also consider the impact of the socioeconomic status of individuals who experience perceived discrimination on their oral health behavior.

- **Response**: Thank you for the comment. The following sentence was added to the manuscript.
- **Revised Text**: Furthermore, it is also important to consider the socioeconomic status of individuals who experience perceived discrimination on their oral health behaviour.

What does the pronoun “those” indicate? Persons with perceived racial discrimination?

- **Response:** Thanks for the comment. We have changed “Those” to “adolescents**”.**
- **Revised text:** Thus, assessment of self-efficacy is beneficial and useful in dental practice to identify adolescents with low self-efficacy.

“Behavioral interventions…”. The present study has no data for the behavioral interventions. Thus, the statement may be excessive discussion.

- **Response:** Thanks for the comment. The following sentences were removed from the manuscript.
- **Revised text: “**Behavioural interventions to increase patient’s self-efficacy may buffer the negative impact of perceived discrimination leading to better adherence of adolescents to healthy behaviours and, consequently, better oral health outcomes” were removed.

Reviewer 2:

Please attach the questionnaire

- **Response:** Thank you for the comment. We have attached the questionnaire.
- **Revised text:** A questionnaire consisting of three sections were completed by adolescents (S1 table).

Indicate the study's design in the abstract

- **Response:** Thank you for the comment. We have added “cross-sectional” to the abstract.

Provide a specific definition of dental health/oral health

- **Response:** Thank you for the comment. A specific definition of oral health has been added to manuscript.
- **Revised text:**

According to Baju et al. (2017) oral health is defined as the well-being and ability to perform functions such as eat, talk, and smile.

You have considered only the frequency of toothbrushing in your study, so how about timing, duration, and method? Why were they not evaluated in the questionnaire?

- **Response:** Thank you for the comment. While all aspects of toothbrushing practices are important, we only included the frequency, which is the most common variable used in oral health studies, to avoid making the questionnaire too long.

Please describe analytical methods taking account of the sampling strategy

- **Response:** Thank you for the comment. Our sampling strategy was added to the text.

**Revised text**: We used a convenience sampling approach and almost all adolescents approached completed the survey.

In the discussion it's better to give more interpretation of results and discuss the generalizability of your results.

- **Response:** Thank you for the comment. More interpretation and generalizability of the results were added to the discussion.
- **Revised text:** Furthermore, it is also important to consider the socioeconomic status of individuals who experience perceived discrimination on their oral health behaviour. The results of this study are not generalizable to the whole adolescent population because the participants were recruited from the University dental clinics, who could have a more favourable attitude towards oral health and dental visits and less barrier to access dental care.

Reviewer 3:

The sample size calculation method should be indicated in details.

- **Response:** Thank you for the comment. We have included the sample size calculation in the manuscript.
- **Revised text:** Given the average yearly number of visits at the university dental clinic, with 95% confidence interval and 5% margin of error desired sample size was 278, however 252 patients agreed to complete the questionnaires. Which is close to the ideal number.

The sampling technique used to recruit participants needs to be more explained: how was the sampling performed?

- **Response**: Thank you for the comment. more information regarding the sampling technique was added to the text.

**Revised text:** We used a convenience sampling approach and almost all adolescents approached completed the survey.
